# Supplementary material for: Intercalated disc protein Xinβ is required for Hippo-YAP signaling in the heart
Source: Nat Commun. 2020 Sep 16;11:4666. doi: 10.1038/s41467-020-18379-8 (PMC7494909; doi:10.1038/s41467-020-18379-8)
Supplement: Supplementary file 1 — Supplementary Information [file 41467_2020_18379_MOESM1_ESM.pdf]

Supplementary Information

Intercalated disc protein Xinβ is required for Hippo-YAP signaling in the heart

Haipeng Guo[1,2], Yao Wei Lu[1], Zhiqiang Lin[1,3], Zhan-Peng Huang[1,4], Jianming Liu[1], Yi Wang[1], Hee Young Seok[1,5], Xiaoyun Hu[1], Qing Ma[1], Kathryn Li[1], Jan Kyselovic[6], Qingchuan Wang[7,8], Jenny L-C. Lin[7], Jim J-C. Lin[7], Douglas B. Cowan[1], Francisco Naya[9], Yuguo Chen[2], William T. Pu[1,10], and Da-Zhi Wang[1,10,*]

*Correspondence:

Da-Zhi Wang, Ph.D.: Da-Zhi.Wang@childrens.harvard.edu
Boston Children's Hospital, 320 Longwood Avenue, Enders 1262, Boston, MA 02115 USA

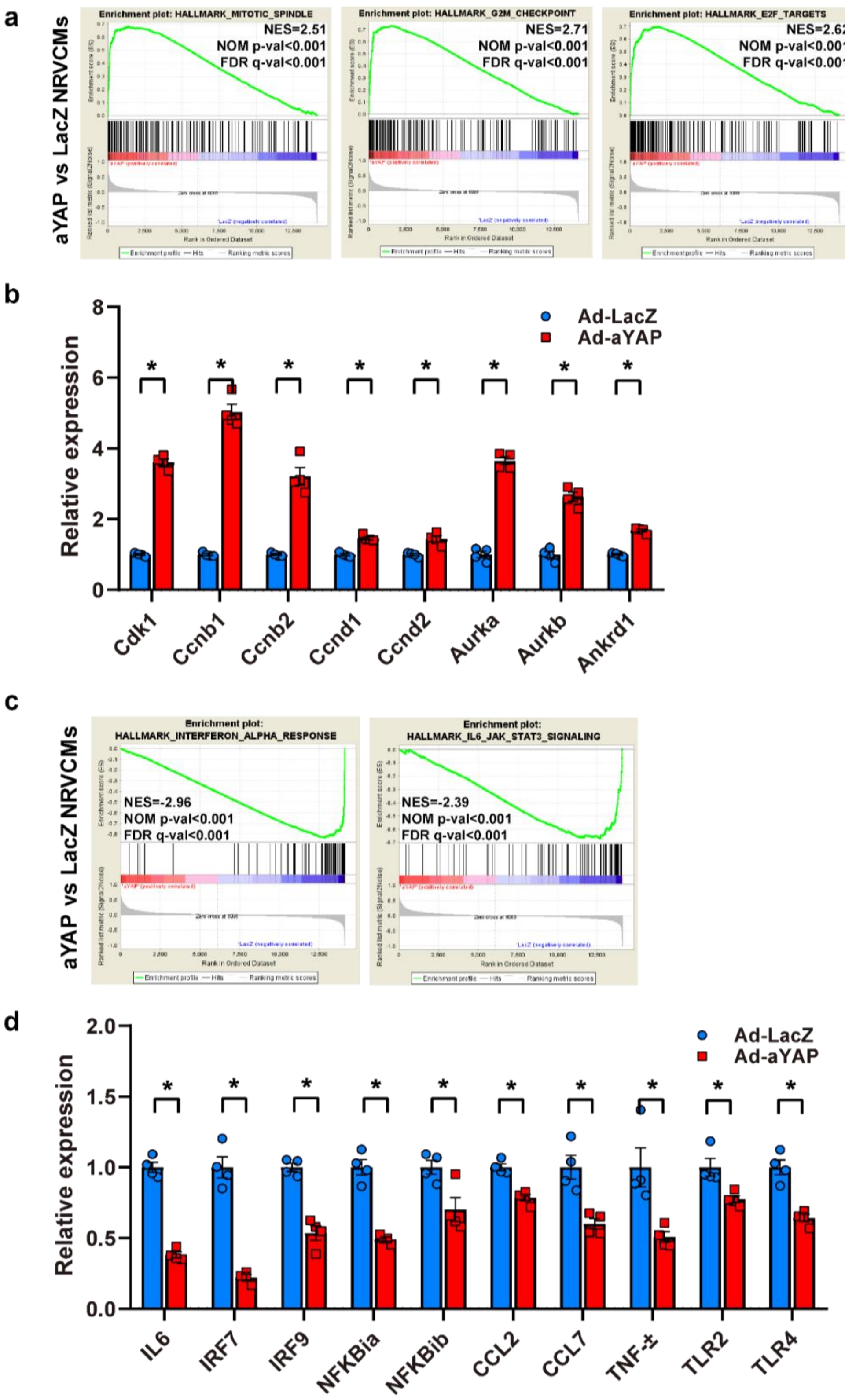

Supplementary Figure 1. Loss of Xinβ results in alteration of the Hippo-YAP signaling pathway in the heart. a, Enrichment plot showing upregulation of genes related to the mitotic spindle, G2M checkpoint, and E2F targets in Ad-*aYAP*-treated cardiomyocytes. **b**, qRT-PCR of cell cycle related genes in Ad-*aYAP* cardiomyocytes. N = 4 biologically independent samples, asterisk P<0.05. **c**, Enrichment plot showing downregulation of genes related to the interferon-α response and IL6-JAK-STAT3 signaling in Ad-*aYAP*-treated cardiomyocytes. **d**, qRT-PCR of innate immune response genes in Ad-*aYAP*-treated cardiomyocytes. N = 4 biologically independent samples, asterisk P<0.05. Exact P-values can be found in the Source Data.

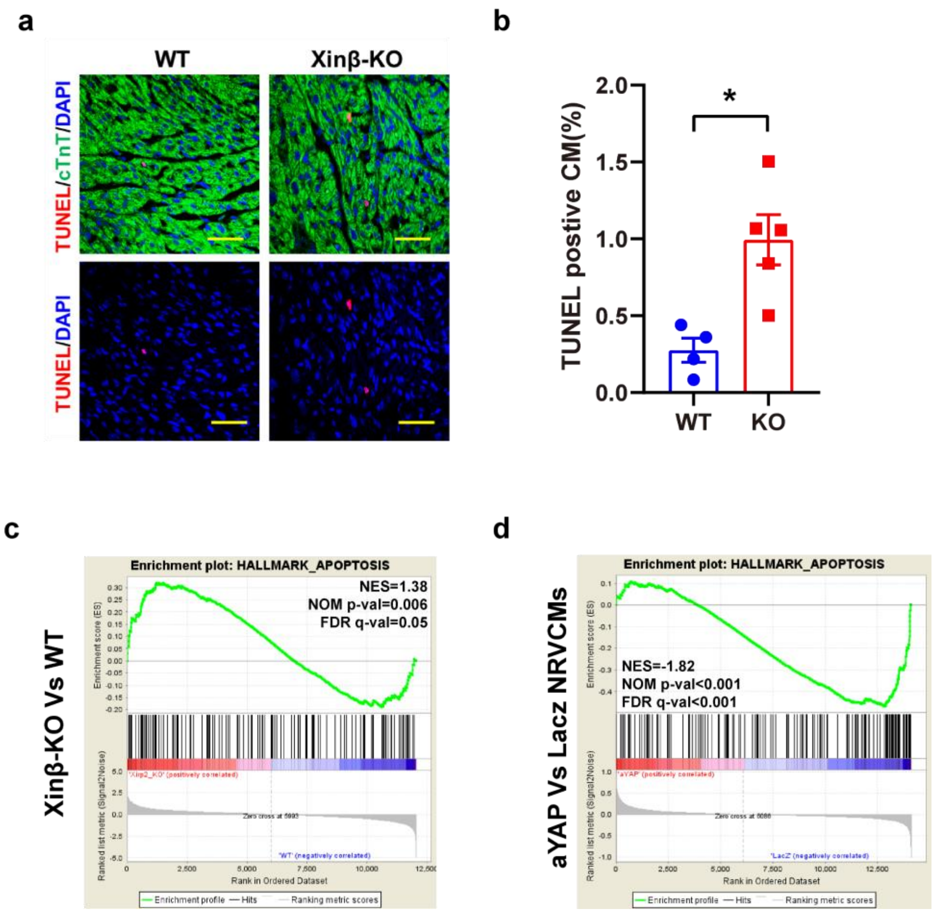

Supplementary Figure 2. Increased apoptosis in cardiomyocytes of Xinβ^{KO} hearts. a, TUNEL assays in P7.5 *Xinβ*^{KO} and control hearts. DAPI marks nuclei, cTnT labels cardiomyocytes. Scale bars = 100 µm. **b,** Quantification of TUNEL staining data. N = 5 biologically independent samples and 4 biologically independent samples, respectively, asterisk $P<0.05$. **c,** Enrichment plot showing upregulation of genes related to apoptosis in P7.5 *Xinβ*^{KO} hearts. **d,** Enrichment plot showing downregulation of genes related to apoptosis in Ad-*aYAP*-treated cardiomyocytes. Exact P-values can be found in the Source Data.

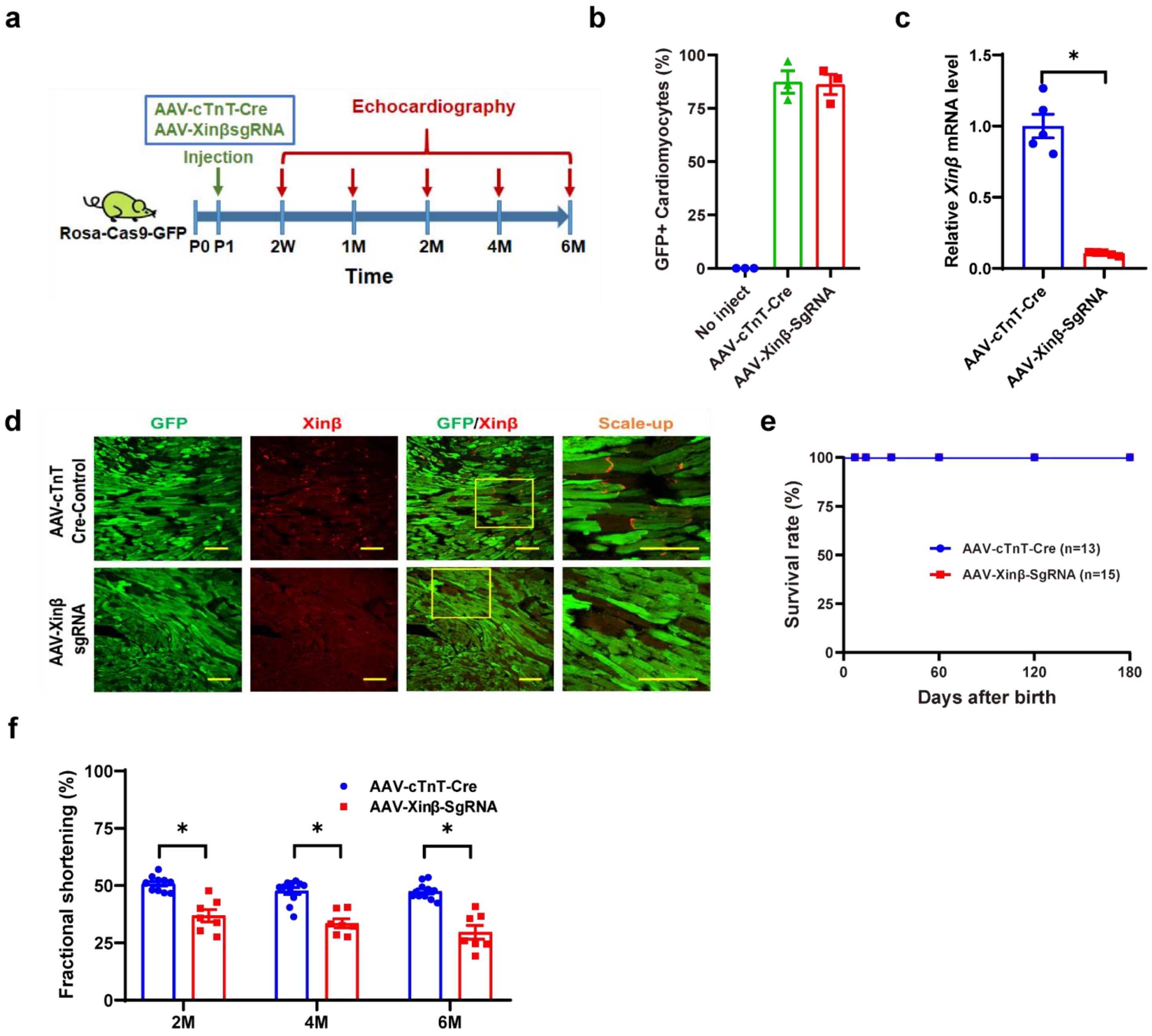

Supplementary Figure 3. AAV9-CAS strategy to generate a Xinβ mutation in adult mice. a, Schematic diagram of the mutation strategy. **b**, Quantification of GFP positive cardiomyocytes from AAV-*cTnT-Cre* or AAV-*Xinβ*^{sgRNA} injected mice. N = 3 biologically independent samples, asterisk P<0.05. **c**, qRT-PCR quantification of *Xinβ* expression in the hearts of AAV-*cTnT-Cre* or AAV-*Xinβ*^{sgRNA} injected mice. N = 5 biologically independent samples, asterisk P<0.05. **d**, Immunohistochemical staining to detect *Xinβ* expression in adult hearts from AAV-*cTnT-Cre* or AAV-*Xinβ*^{sgRNA} injected mice. Scale bars = 20 µm. **e**, Survival curve of AAV-*cTnT-Cre* or AAV-*Xinβ*^{sgRNA} injected mice. N = 13 or 15 biologically independent samples, respectively. **f,** Cardiac fractional shortening (FS%) in AAV-*cTnT-Cre* or AAV-*Xinβ*^{sgRNA} injected mice at 2, 4, and 6 months (M) of age. N = 11 and 7 biologically independent samples, respectively, asterisk P<0.05. Exact P-values can be found in the Source Data.

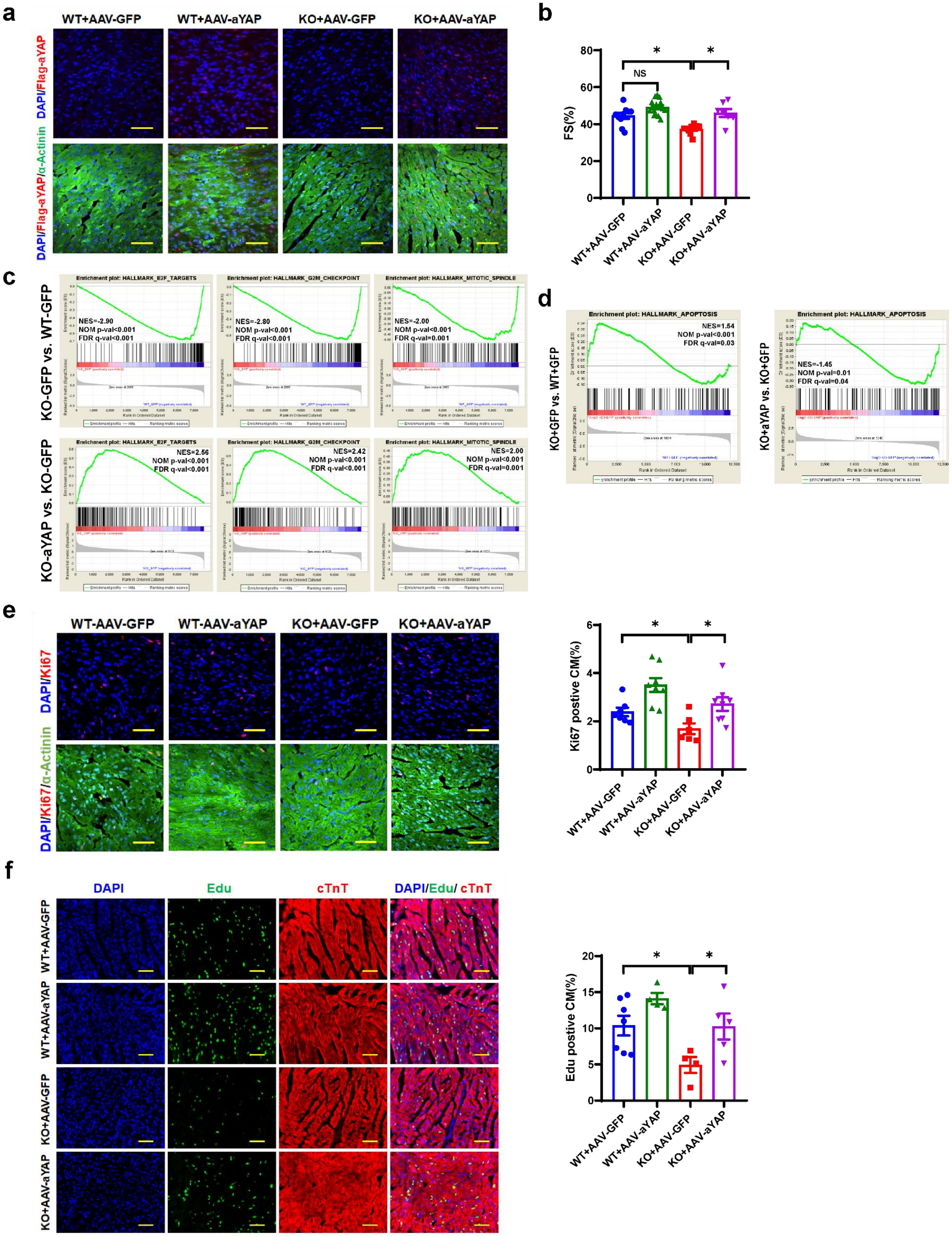

a
WT+AAV-GFP
WT+AAV-aYAP
KO+AAV-GFP
KO+AAV-aYAP
DAPI/Flag-aYAP
DAPI/Flag-aYAP/α-Actinin
b
FS(%)
NS
*
*
80
60
40
20
0
WT+AAV-GFP
WT+AAV-aYAP
KO+AAV-GFP
KO+AAV-aYAP
c
KO-GFP vs. WT-GFP
KO-aYAP vs. KO-GFP
Enrichment plot: HALLMARK_E2F_TARGETS
Enrichment plot: HALLMARK_G2M_CHECKPOINT
Enrichment plot: HALLMARK_MITOTIC_SPINDLE
NES=-2.90
NOM p-val<0.001
FDR q-val<0.001
NES=-2.80
NOM p-val<0.001
FDR q-val<0.001
NES=-2.00
NOM p-val<0.001
FDR q-val=0.001
NES=2.56
NOM p-val<0.001
FDR q-val<0.001
NES=2.42
NOM p-val<0.001
FDR q-val<0.001
NES=2.00
NOM p-val<0.001
FDR q-val=0.001
d
KO+GFP vs. WT+GFP
KO+aYAP vs. KO+GFP
Enrichment plot: HALLMARK_APOPTOSIS
NES=1.54
NOM p-val<0.001
FDR q-val=0.03
NES=-1.45
NOM p-val=0.01
FDR q-val=0.04
e
WT-AAV-GFP
WT-AAV-aYAP
KO+AAV-GFP
KO+AAV-aYAP
DAPI/Ki67
DAPI/Ki67/α-Actinin
Ki67 postive CM(%)
*
*
6
4
2
0
WT+AAV-GFP
WT+AAV-aYAP
KO+AAV-GFP
KO+AAV-aYAP
f
DAPI
Edu
cTnT
DAPI/Edu/ cTnT
WT+AAV-GFP
WT+AAV-aYAP
KO+AAV-GFP
KO+AAV-aYAP
Edu postive CM(%)
*
*
20
15
10
5
0
WT+AAV-GFP
WT+AAV-aYAP
KO+AAV-GFP
KO+AAV-aYAP

Supplementary Figure 4. Heart-specific YAP^{S127A} overexpression rescues cardiac gene expression in Xinβ^{KO} mice. a, Immunohistochemical staining to detect expression of Flag-tagged YAP in the hearts of P7.5 *Xinβ*^{KO} and control mice injected with AAV-*aYAP* (*YAP*^{S127A}) or control AAV-*GFP*. DAPI marks nuclei, α-actinin labels cardiomyocytes. Scale bars = 40 µm. **b,** Fractional shortening (FS%) of P7.5 *Xinβ*^{KO} and control mice injected with AAV-*YAP* or control AAV-*GFP*. N = 7 biologically independent samples (*Xinβ*^{KO}) and 15 or 10 biologically independent samples (control), respectively, asterisk P<0.05. **c,** Enrichment plot showing restored expression of genes related to cell proliferation in P7.5 *Xinβ*^{KO} mice after injection of AAV-*aYAP*. **d,** Enrichment plot showing inhibition of the expression of genes related to apoptosis in P7.5 *Xinβ*^{KO} mice after injection of AAV-*aYAP*. **e,** Ki67 staining of hearts from P7.5 *Xinβ*^{KO} and control mice injected with AAV-*aYAP* or control AAV-*GFP*. DAPI marks nuclei, α-actinin labels cardiomyocytes. Quantification is shown in the right panel. Scale bars = 40 µm, N = 6 biologically independent samples (*Xinβ*^{KO} AAV-*GFP*) or 7 biologically independent samples (*Xinβ*^{KO} AAV-*aYAP*) and 7 biologically independent samples (control AAV-*aYAP* and AAV-*GFP*), respectively, asterisk P<0.05. **f,** EdU incorporation in hearts from P7.5 *Xinβ*^{KO} and control mice injected with AAV-*aYAP* or control AAV-*GFP*. DAPI marks nuclei, cTnT labels cardiomyocytes. Quantification is shown in the right panel. Scale bars = 40 µm. N = 5 biologically independent samples (*Xinβ*^{KO} AAV-*aYAP*), 4 (*Xinβ*^{KO} AAV-*GFP*), 4 biologically independent samples (control AAV-*aYAP*), and 7 biologically independent samples (control AAV-*GFP*), asterisk P<0.05. Exact P-values can be found in the Source Data.

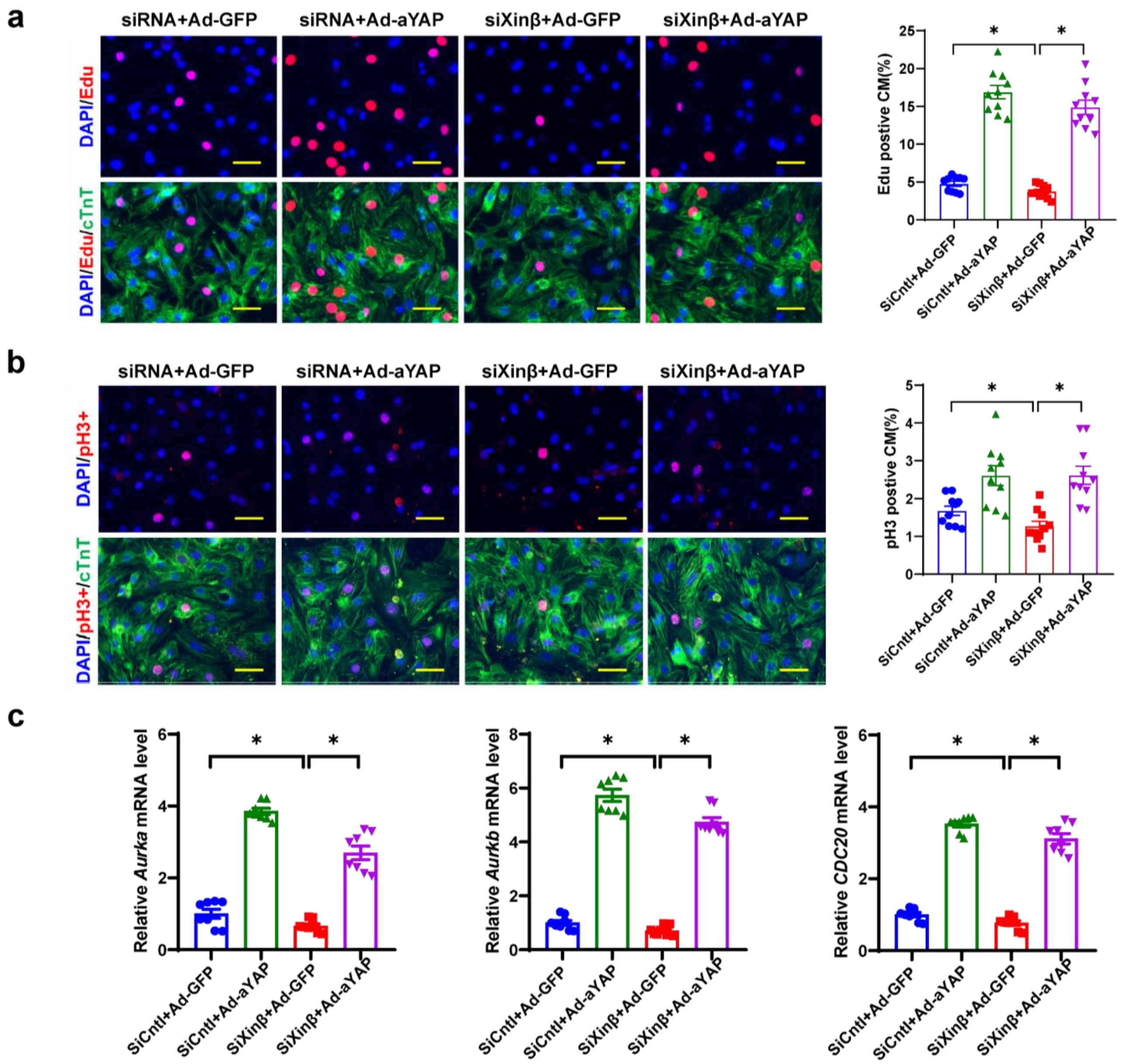

Supplementary Figure 5. Regulation of cardiomyocyte proliferation by Xinβ and YAP. a, Primary neonatal (P1) rat ventricular cardiomyocytes (NRVMs) were transfected with siRNA against *Xinβ* (si*Xinβ*) or control siRNA. Cells were transduced with adenovirus expressing either an active YAP (Ad-*aYAP*, which contains the S127A mutation [*YAP*^{S127A}]) or Ad-*GFP*. Cells were incubated with EdU (10 μM). After one day, cultures were fixed and stained with antibodies for EdU. Cardiac troponin T (cTNT) marks cardiomyocytes. DAPI stains nuclei. Scale bars = 70 μm. Quantification of percentages of EdU$^+$ cardiomyocytes is presented in the right panel. N = 10 biologically independent samples, asterisk P<0.05. **b,** Primary neonatal (P1) rat cardiomyocytes were transfected with siRNA against *Xinβ* (si*Xinβ*) or control siRNA. Cells were transduced with an active YAP (Ad-*aYAP*) or control (Ad-*GFP*). After one day, cultures were fixed and stained with antibodies for phospho-histone H3 (pH3). Cardiac troponin T (cTNT) marks cardiomyocytes. DAPI stains nuclei. Scale bars = 70 μm. Quantification of percentages of pH3$^+$ cardiomyocytes is presented in the right panel. N = 10 biologically independent samples, asterisk P < 0.05. **c,** Quantitative RT-PCR (qRT-PCR) analyses of the expression of cell proliferation marker genes in NRVMs cultured in the above conditions. N = 8 biologically independent samples, asterisk P<0.05. Exact P-values can be found in the Source Data.
