# Supplementary material for: Intercalated disc protein Xinβ is required for Hippo-YAP signaling in the heart
Source: Nat Commun. 2020 Sep 16;11:4666. doi: 10.1038/s41467-020-18379-8 (PMC7494909; doi:10.1038/s41467-020-18379-8)
Supplement: Supplementary file 3 — Description of Additional Supplementary Files [file 41467_2020_18379_MOESM3_ESM.docx]

**Description of Additional Supplementary Files**

File Name: Supplementary Data 1

Description: List of antibodies (Table I) and primers (Table II) used in this study. Table I contains information about the antibody source and the dilutions used for Western Blots (WB), immunoprecipitations (IP), and immunofluorescence staining (IF). Table II lists the forward and reverse direction DNA sequences of the primers.
