# Supplementary material for: Intercalated disc protein Xinβ is required for Hippo-YAP signaling in the heart
Source: Nat Commun. 2020 Sep 16;11:4666. doi: 10.1038/s41467-020-18379-8 (PMC7494909; doi:10.1038/s41467-020-18379-8)
Supplement: Supplementary file 4 — Supplementary Data 1 [file 41467_2020_18379_MOESM4_ESM.docx]

**Supplementary Data 1**

**Table I. Antibodies**

| **Antigen** | **Company** | **Catalog** | **Origin** | **Working dilution** |
| --- | --- | --- | --- | --- |
| T-YAP1 | Cell Signaling Technology | 14074 | Rabbit | 1:500 for WB  1:100 for IF |
| P-YAP1 | Cell Signaling Technology | 13008 | Rabbit | 1:500 for WB |
| T-AKT | Cell Signaling Technology | 4691 | Rabbit | 1:1000 for WB |
| P-AKT | Cell Signaling Technology | 4060 | Rabbit | 1:1000 for WB |
| T-GSK3β | Cell Signaling Technology | 12456 | Rabbit | 1:1000 for WB |
| P- GSK3β | Cell Signaling Technology | 9336 | Rabbit | 1:1000 for WB |
| T-ERK1/2 | Cell Signaling Technology | 9101 | Rabbit | 1:1000 for WB |
| P-ERK1/2 | Cell Signaling Technology | 4370 | Rabbit | 1:1000 for WB |
| T-NF2 | Santa Cruz | SC-55575 | Mouse | 1:50 for IF |
| T-NF2 | Cell Signaling Technology | 12888 | Rabbit | 1:500 for WB  1:100 for IF |
| P-NF2 | Cell Signaling Technology | 9163 | Rabbit | 1:500 for WB  1:100 for IF |
| T-MST1 | Cell Signaling Technology | 3682 | Rabbit | 1:500 for WB |
| P-MST1 | Sigma-Aldrich | SAB4504042 | Rabbit | 1:500 for WB  1:100 for IF |
| T-Lats1 | Cell Signaling Technology | 3477 | Rabbit | 1:200 for WB |
| P-Lats1 | Cell Signaling Technology | 8654 | Rabbit | 1:200 for WB  1:100 for IF |
| pH3 | Sigma-Aldrich | 06-570 | Rabbit | 1:200 for IF |
| Ki67 | Abcam | ab16667 | Rabbit | 1:200 for IF |
| CX43 | Cell Signaling Technology | 3512 | Rabbit | 1:200 for IF |
| N-Cadherin | Invitrogen | 33-3900 | Mouse | 1:200 for IF |
| Flag | Sigma-Aldrich | F7425 | Rabbit | 1:2000 for WB  1:200 for IF |
| GFP | ROCKLAND | 600-101-215 | Goat | 1:2000 for WB  1:200 for IF |
| a-Actinin | Sigma-Aldrich | A7811 | Mouse | 1:200 for IF |
| cTnT | ThermoFisher | MA5-12960 | Mouse | 1:200 for IF |
| β-Tublin | Abcam | ab131205 | Mouse | 1:5000 for WB |
| GAPDH | Fitzgerald | 10-1501 | Mouse | 1:5000 for WB |

| Antibody Name | Clone | Origin | Working Dilution |
| --- | --- | --- | --- |
| Rabbit anti-Xinβ | U1040B | Rabbit | 1:200 for IP or WB; 1:100 for IF |

| Name | Company | Origin | Working Dilution | Catalog | Lot |
| --- | --- | --- | --- | --- | --- |
| Alexa Fluor 488-goat anti-mouse IgG (H+L) | Invitrogen | Goat | 1:800 for IF | A11029 | 1911843 |
| Alexa Fluor 594-goat anti-rabbit IgG (H+L) | Invitrogen | Goat | 1:800 for IF | A11012 | 1892265 |
| Alexa Fluor 647-goat anti-rabbit IgG (H+L) | Invitrogen | Goat | 1:800 for IF | A21245 | 2098544 |
| Alexa Fluor 594-donkey anti-goat IgG (H+L) | Invitrogen | Donkey | 1:800 for IF | A11058 | 54258A |
| Alexa Fluor 594-goat anti-mouse IgG (H+L) | Invitrogen | Goat | 1:800 for IF | A21135 | 99E3-1 |
| IRDye 800CW goat anti-Mouse | LI-COR | Mouse | 1:10000 for WB | 926-68070 | C90219-05 |
| IRDye 800CW goat anti-rabbit | LI-COR | Rabbit | 1:10000 for WB | 926-32211 | C81210-05 |

**Table II. Primers**

| **Gene** | **Forward (5’-3’)** | **Reverse (5’-3’)** |
| --- | --- | --- |
| *mXinβ* | *AACCTGCAACGCCTTTTCT* | *TCTTGACTGCTGTGGATTGC* |
| *rXinβ* | *CACAGAACTGATGTCTTCAAAGC* | *GGAATCTCTTCGTCTTCTGGTG* |
| *hXinβ* | *ATTGAACGCTTTTCCATTGC* | *CGCTCAATTTCCACTTCCTT* |
| *m/hYAP1* | *GACCCTCGTTTTGCCATGAA* | *ATTGTTCTCAATTCCTGAGAC* |
| *mYAP1* | *CCCAGGAGAAGACACTGCAT* | *AAATGCTCCAAAATGTCAGGA* |
| *rYAP1* | *CCTTTGAGATCCCTGATGATG* | *TCCTGCCATGTTGTTGTCTG* |
| *hYAP1* | *AGAACTGCTTCGGCAGGA* | *TGGATTTTGAGTCCCACCAT* |
| *mTEAD1* | *TCATTCCAAGCTGAAGGTAACA* | *AGACGATCTGGGCTGATGAC* |
| *rTEAD1* | *CAAACTGAGGACGGGAAAGA* | *TGGCAAGAACCTGAATGTGA* |
| *mCTNNB1* | *TGCAGATCTTGGACTGGACA* | *AAGAACGGTAGCTGGGATCA* |
| *rCTNNB1* | *GCTTACGGCAATCAGGAAAG* | *TCAGCACTCTGCTTGTGGTC* |
| *mFSP1* | *GGAGCTGCCTAGCTTCCTG* | *TCCTGGAAGTCAACTTCATTGTC* |
| *mPOSTN* | *TCCACCTGATTGATGAAGTCC* | *GTCGGTGAAAGTGGTTTGCT* |
| *mcTnT* | *AGCCCACATGCCTGCTTA* | *ACATGCTCTCGGCTCTCC* |
| *mNppa* | *GGGTCTTCAGGAGGATCTGA* | *CCTCATCTTCTACCGGCATC* |
| *mNppb* | *GTCAGTCGTTTGGGCTGTAAC* | *AGACCCAGGCAGAGTCAGAA* |
| *mCX43* | *GTGCCGGCTTCACTTTCA* | *GGAGTAGGCTTGGACCTTGTC* |
| *mN-cadherin* | *CCTCCATGTGCCGGATAG* | *CACCAGAAGCCTCCACAGAC* |
| *mFas* | *CAAGTGCAAGTGCAAACCAG* | *GGGTTCCATGTTCACACGA* |
| *mPmaip1* | *CAGATGCCTGGGAAGTCG* | *TGAGCACACTCGTCCTTCAA* |
| *mMDM2* | *TGTGTGAGCTGAGGGAGATG* | *CACTTACGCCATCGTCAAGA* |
| *mBax* | *GTGAGCGGCTGCTTGTCT* | *GTGGGGGTCCCGAAGTAG* |
| *mBcl-2* | *GTACCTGAACCGGCATCTG* | *GCTGAGCAGGGTCTTCAGAG* |
| *mPERP* | *GACCCCAGATGCTTGTTTTC* | *ACCAGGGAGATGATCTGGAA* |
| *mTnfrsf10b* | *CCCTGAGATCTGCCAGTCAT* | *TTTCTCTGGGGGTACAGGAA* |
| *mAXL* | *GCTCACCCACTGCAACCT* | *GGGTCTTCAGGAGGATCTGA* |
| *mMARCKs* | *GTCGCCTTCCAAAGCAAAT* | *CTTGCAGCTCCTCCTTGG* |
| *mCDK1* | *TTTCGGCCTTGCCAGAGCGTT* | *GTGGAGTAGCGAGCCGAGCC* |
| *rCDK1* | *TCATGGATTCTTCGCTCGTT* | *TCGGGAGTGACAAAACACAA* |
| *mCyclinA1* | *GCCTTCACCATTCATGTGGAT* | *TTGCTCCGGGTAAAGAGACAG* |
| *rCyclinA1* | *GAGGGAAATTGCAGCTTGTC* | *GGGCGGGTATATCTCTTCGT* |
| *mCyclinB1* | *AAGGTGCCTGTGTGTGAACC* | *GTCAGCCCCATCATCTGCG* |
| *rCyclinB1* | *CCTTGACAACGGTGAATGG* | *TCTTAGCCAGGTGCTGCAT* |
| *mCyclinD1* | *CATCCATGCGGAAAATCG* | *CAGGCGGCTCTTCTTCAA* |
| *rCyclinD1* | *GCACAACGCACTTTCTTTCC* | *TCCAGAAGGGCTTCAATCTG* |
| *mCDC20* | *ACATCAAGGCGCTGTCAAG* | *AATGTGCCGGTCACTGGT* |
| *rCDC20* | *GAGTGCTGTGGATGTGCATT* | *GCTCCTTATAGTGGGGAGACC* |
| *mAurka* | *GGGTGGTCGGTGCATGCTCCA* | *GCCTCGAAAGGAGGCATCCCCACTA* |
| *rAurka* | *GTCTGTGCATGCTCCGTCT* | *TCCGACCTTCAATCATCTCC* |
| *mAurkb* | *ATTGCAGACTTTGGCTGGTC* | *AATCATCTCTGGGGGCAGAT* |
| *rAurkb* | *CTTTGGCTGGTCTGTGCAT* | *CTGGGGGCAGATAGTCCAG* |
| *mAnkrd1* | *CGGACCTCAAGGTCAAGAAC* | *TGAGGCTGTCGAATATTGCTT* |
| *mMyh6* | *CTCTGGATTGGTCTCCCAGC* | *GTCATTCTGTCACTCAAACTCTGG* |
| *mMyh7* | *CTCAGAGCTCAAGCGGGATA* | *CCAGCCATCTCCTCTGTCA* |
| *mCol1a1* | *CCGCTGGTCAAGATGGTC* | *CTCCAGCCTTTCCAGGTTCT* |
| *mFN1* | *CTTTGTGGTCTCATGGGTCTC* | *AGCAGGTCAGGAATGTTCAC* |
| *mβ-actin* | *CTAAGGCCAACCGTGAAAAG* | *ACCAGAGGCATACAGGGACA* |
| *rβ-actin* | *TGCCCTAGACTTCGAGCAAG* | *GGCAGCTCATAGCTCTTCTCC* |
| *18S* | *GCAATTATTCCCCATGAACG* | *GGGACTTAATCAACGCAAGC* |
