# Supplementary material for: Intercalated disc protein Xinβ is required for Hippo-YAP signaling in the heart
Source: Nat Commun. 2020 Sep 16;11:4666. doi: 10.1038/s41467-020-18379-8 (PMC7494909; doi:10.1038/s41467-020-18379-8)
Supplement: Supplementary file 5 — Reporting Summary [file 41467_2020_18379_MOESM5_ESM.pdf]

nature research

Corresponding author(s): Da-Zhi Wang, Ph.D.

Last updated by author(s): Jul 22, 2020

Reporting Summary

Nature Research wishes to improve the reproducibility of the work that we publish. This form provides structure for consistency and transparency in reporting. For further information on Nature Research policies, see our Editorial Policies and the Editorial Policy Checklist.

Statistics

For all statistical analyses, confirm that the following items are present in the figure legend, table legend, main text, or Methods section.

n/a	Confirmed	
☐	☒	The exact sample size (*n*) for each experimental group/condition, given as a discrete number and unit of measurement
☐	☒	A statement on whether measurements were taken from distinct samples or whether the same sample was measured repeatedly
☐	☒	The statistical test(s) used AND whether they are one- or two-sided<br>*Only common tests should be described solely by name; describe more complex techniques in the Methods section.*
☐	☒	A description of all covariates tested
☐	☒	A description of any assumptions or corrections, such as tests of normality and adjustment for multiple comparisons
☐	☒	A full description of the statistical parameters including central tendency (e.g. means) or other basic estimates (e.g. regression coefficient) AND variation (e.g. standard deviation) or associated estimates of uncertainty (e.g. confidence intervals)
☐	☒	For null hypothesis testing, the test statistic (e.g. *F*, *t*, *r*) with confidence intervals, effect sizes, degrees of freedom and *P* value noted<br>*Give P values as exact values whenever suitable.*
☒	☐	For Bayesian analysis, information on the choice of priors and Markov chain Monte Carlo settings
☐	☒	For hierarchical and complex designs, identification of the appropriate level for tests and full reporting of outcomes
☐	☒	Estimates of effect sizes (e.g. Cohen's *d*, Pearson's *r*), indicating how they were calculated

Our web collection on statistics for biologists contains articles on many of the points above.

Software and code

Policy information about availability of computer code

Data collection	No software was used for data collection.
Data analysis	For RNA-seq data analysis, FASTQ files were extracted and the TruSeq sequencing adapters and low quality reads were removed from FASTQ files with Cutadapt v.2.3. The cleaned FASTQ files were quality checked using FastQC (Babraham Bioinformatics), Transcript assembly was performed using StringTie (v.1.3.4) with the annotated transcriptome as a reference. The assembled transcriptomes were quantified using prepDE.py script provided by the StringTie developer to generate gene matrix files. EdgeR (v.3.26.1) was used to compute counts per million (CPM) as a normalized measurement for gene expression. Differentially expressed genes were tested using the Fisher's exact test and multiplicity correction was performed using the Benjamini-Hochberg method on the P-values to control the false discovery rate (FDR). Differentially regulated genes with FDR values < 0.05 were considered significant. We defined expressed genes as those that have expression in at least half of all samples. Expressed genes were subjected to Principal Component Analysis (PCA). Principal components 1 and 2 were plotted in 2-D coordinates. Gene Set Enrichment Analysis (GSEA) was performed on expressed genes according to the software manual. Gene sets with a nominal P-value of < 0.05 and a FDR of < 0.25 were considered significant. All expressed genes were Log2 transformed, centered, and unsupervised hierarchical clustering was performed using k-mean clustering method with Cluster 3.0 software. Java Treeview (v.3.0) was used to visualize the clustered heatmaps. Differentially regulated genes with a Benjamini-Hochberg FDR < 0.05 were considered significant. Microscopic images of histological sections were quantified with ImageJ v1.52 software. All relevant data related to this manuscript are available upon reasonable request from the authors. The RNA-seq data described in this study has been deposited to the Gene Expression Omnibus (GSE) with the accession number GSE149647 (https://www.ncbi.nlm.nih.gov/gds/?term=GSE149647[Accession]). Source Data are provided with this paper. Original un-cropped western blots and raw numbers used for the statistics presented in the Figures and Supplementary Information are contained in the Source Data. A list of the antibodies and primer sequences used in this study are provided in Supplementary Data File 1 (Tables I and II).

For manuscripts utilizing custom algorithms or software that are central to the research but not yet described in published literature, software must be made available to editors and reviewers. We strongly encourage code deposition in a community repository (e.g. GitHub). See the Nature Research guidelines for submitting code & software for further information.

Data

Policy information about availability of data

All manuscripts must include a data availability statement. This statement should provide the following information, where applicable:

- Accession codes, unique identifiers, or web links for publicly available datasets
- A list of figures that have associated raw data
- A description of any restrictions on data availability

All relevant data related to this manuscript are available on request from the authors on reasonable request. FASTQ files were aligned to the mouse genome (Esembl GRCm38 genome obtained from GENCODE) using HISAT2 (v.2.1.0). The accession number for the RNA-seq genomic data described in this study is GSE149647 (https://www.ncbi.nlm.nih.gov/gds/?term=GSE149647[Accession]). Original un-cropped western blots are provided in Source Data as are the raw numbers used for the statistics presented in the Figures and Supplementary Information A list of the antibodies and primer sequences used in this study are also provided (Supplementary Data File 1). aYAP over-expression in the neonatal rat ventricular cardiomyocyte microarray data was obtained from the Gene Expression Omnibus (GEO) accession number GSE57719 (https://www.ncbi.nlm.nih.gov/gds/?term=GSE57719[Accession]).

Field-specific reporting

Please select the one below that is the best fit for your research. If you are not sure, read the appropriate sections before making your selection.

☒ Life sciences ☐ Behavioural & social sciences ☐ Ecological, evolutionary & environmental sciences

For a reference copy of the document with all sections, see nature.com/documents/nr-reporting-summary-flat.pdf

Life sciences study design

All studies must disclose on these points even when the disclosure is negative.

Sample size	No sample size calculation was performed. We chose the sample sizes based on the literature, which was sufficient to analyze significance between groups. The number of the independent experiments for cell and biological experiments is indicated in the Source Data.
Data exclusions	Sick or diseased animals were excluded. The exclusions were based on criteria from the literature and the recommendation of the veterinarian. The exclusions criteria were unbiasedly applied on the described experiments involving mouse model.
Replication	In each experimental group, 5 mice were included and independent experiments were repeated at least 3 times. For western blotting, we repeated independently conducted experiments at least 3 times. The results of all in vivo experiments were reproducible as shown across multiple animals (exact n values indicated in the Source Data over multiple cohorts. Where possible quantitative PCR, western blotting, immunohistochemistry, and RNA sequencing analysis was performed with at least three independent biological replicates and minimal variation.
Randomization	Animals were randomized into groups with the same genotypes, gender, and age. Mice were bred in-house with groups being populated with mice as soon as they became available. Mice were randomly assigned to groups where applicable. Experimental groups provide a roughly equal mix of males and females.
Blinding	Individuals collecting data or adjudicating outcomes were blinded to the groups or cohorts being analyzed.

Reporting for specific materials, systems and methods

We require information from authors about some types of materials, experimental systems and methods used in many studies. Here, indicate whether each material, system or method listed is relevant to your study. If you are not sure if a list item applies to your research, read the appropriate section before selecting a response.

Materials & experimental systems

n/a	Involved in the study	
☐	☒	Antibodies
☐	☒	Eukaryotic cell lines
☒	☐	Palaeontology and archaeology
☐	☒	Animals and other organisms
☐	☒	Human research participants
☒	☐	Clinical data
☒	☐	Dual use research of concern

Methods

n/a	Involved in the study	
☒	☐	ChIP-seq
☒	☐	Flow cytometry
☒	☐	MRI-based neuroimaging

Antibodies

Antibodies used	The anti-mouse Xinβ (U1040) primary antibody was obtained from the authors of the following article: Am J Physiol Heart Circ Physiol

	293: H2680–H2692. A list of all antibodies (including sources and dilutions used) are provided in Supplementary Data File 1 (Table I).
Validation	Antibodies were validated using western blotting and immunofluorescence staining (immunostaining) in mouse tissue.

Eukaryotic cell lines

Policy information about cell lines

Cell line source(s)	HEK293T cells were obtained from the American Type Culture Collection (CRL-3216).
Authentication	Cells were authenticated by immunostaining, western blot analysis, and gene expression analyses (e.g. qRT-PCR).
Mycoplasma contamination	All cell lines used in this study tested negative for mycoplasma before their use.
Commonly misidentified lines (See ICLAC register)	No commonly misidentified cell lines were used in this study.

Animals and other organisms

Policy information about studies involving animals; ARRIVE guidelines recommended for reporting animal research

Laboratory animals	All animal protocols were approved by the Institutional Animal Care and Use Committee of Boston Children's Hospital. Mice were maintained at temperatures of 65-75°F (~18-23°C) with 40-60% humidity and a light:dark cycle of 12h:12h. Xinβ knockout mice (Xinβ-KO) were described previously (Circ Res 106: 1468-78) and these mice were back-crossed to, and maintained in, a C57BL/6J background. All RosaCas9GFP/Cas9GFP mice were acquired from the Jackson Laboratory (#026175). Both male and female mice at the ages indicated in the manuscript and figures were used.
Wild animals	No wild animals were used in this study.
Field-collected samples	No field-collected samples were used in this study.
Ethics oversight	Ethical oversight was provided by the Institutional Animal Care and Use Committee of Boston Children's Hospital.

Note that full information on the approval of the study protocol must also be provided in the manuscript.

Human research participants

Policy information about studies involving human research participants

Population characteristics	De-identified left ventricle (LV) tissues were taken from patients with terminal-stage heart failure that were indicated for heart transplantation. Ten patients (9 male and 1 female) with dilated cardiomyopathy between the ages of 34 and 62 were used for these studies. In brief, the patient's heart was removed at the time of transplantation, and LV tissue was dissected and snap frozen in liquid nitrogen. We used LV samples from healthy hearts that were not implanted to serve as controls. There was no record linking the subject and the research to avoid the possibility of a breach of confidentiality.
Recruitment	Patients with heart failure were recruited according to the Institutional Review Board (IRB) protocol approved by the Institutional Ethics Committee at the National Institute of Cardiovascular Diseases, Bratislava, Slovakia. Informed consent was sought from each prospective subject or the subject's legally authorized representative in accordance with, and to the extent required by 45 CFR 46.116 and 21 CFR 50.20. In addition, the IRB protocol ensured that informed consent was appropriately documented, in accordance with, and to the extent required by 45 CFR 46.117 and 21 CFR 50.27.
Ethics oversight	Human samples were collected in accordance with the World Medical Association's (WMA) Declaration of Helsinki and procedures were approved by the Institutional Ethics Committee of the National Institute of Cardiovascular Diseases, Bratislava, Slovakia. Patients provided written informed consent prior to tissue collection.

Note that full information on the approval of the study protocol must also be provided in the manuscript.
