# Supplementary figures and images for: Intercalated disc protein Xinβ is required for Hippo-YAP signaling in the heart
Source: Nat Commun. 2020 Sep 16;11:4666. doi: 10.1038/s41467-020-18379-8 (PMC7494909; doi:10.1038/s41467-020-18379-8)

Fig 4a

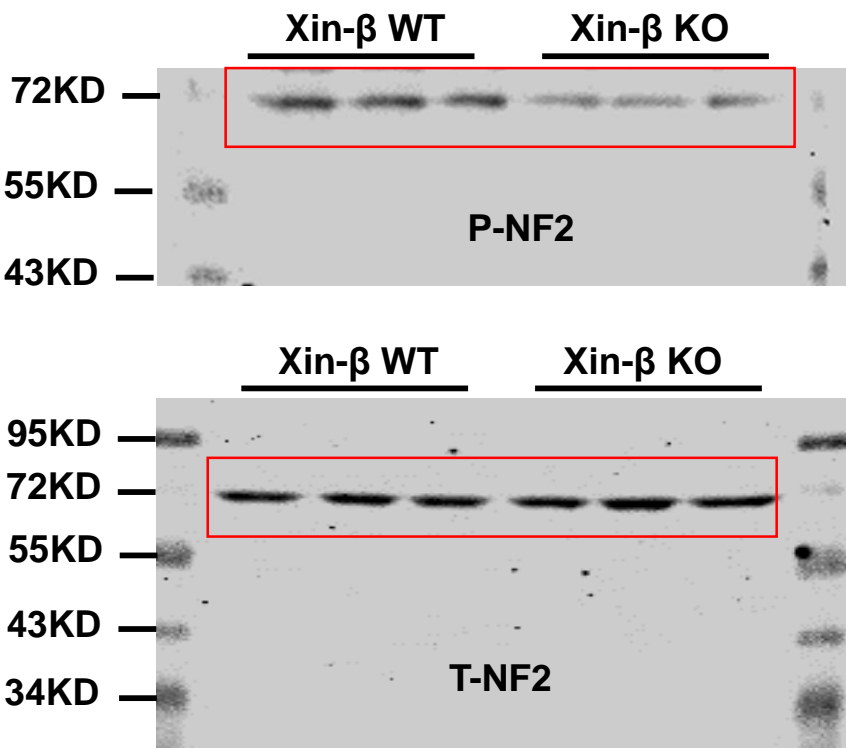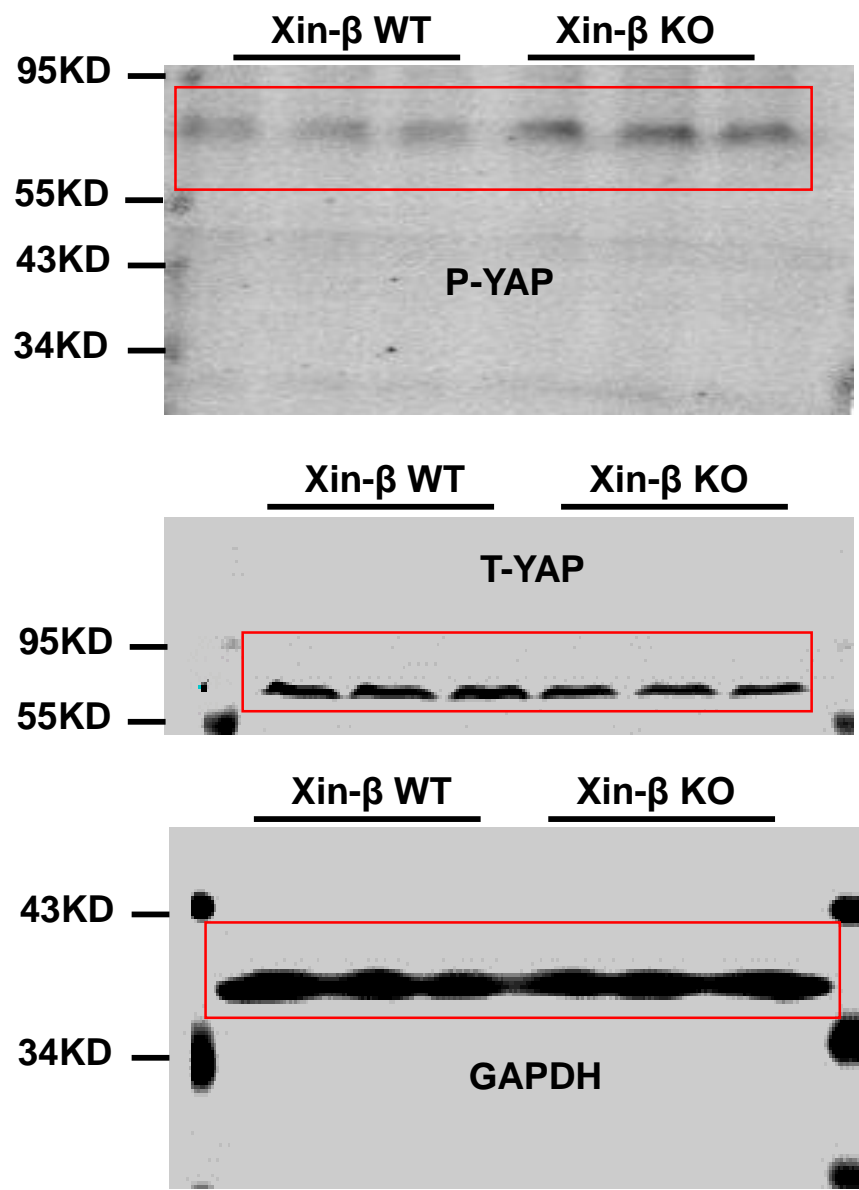

Fig 4f

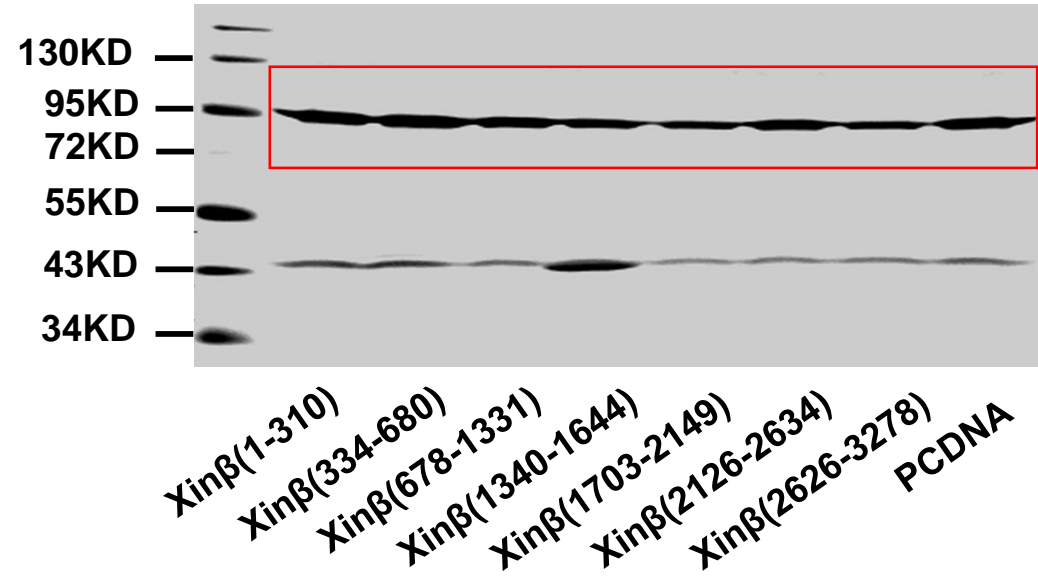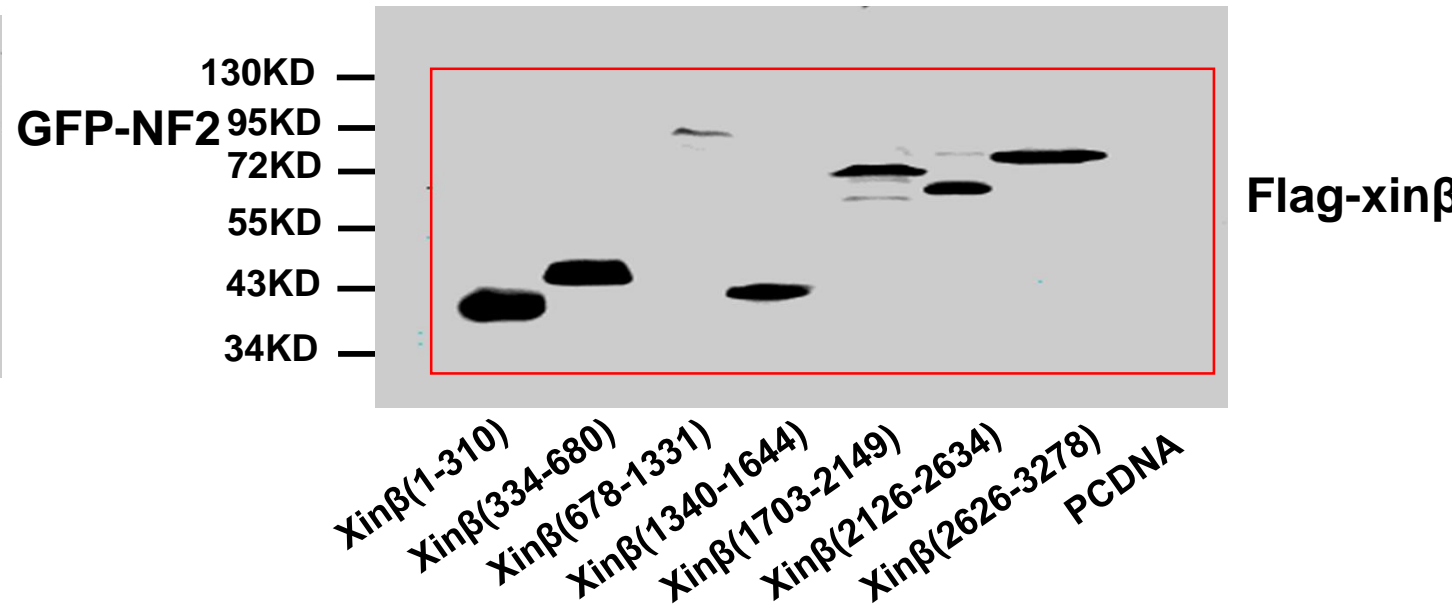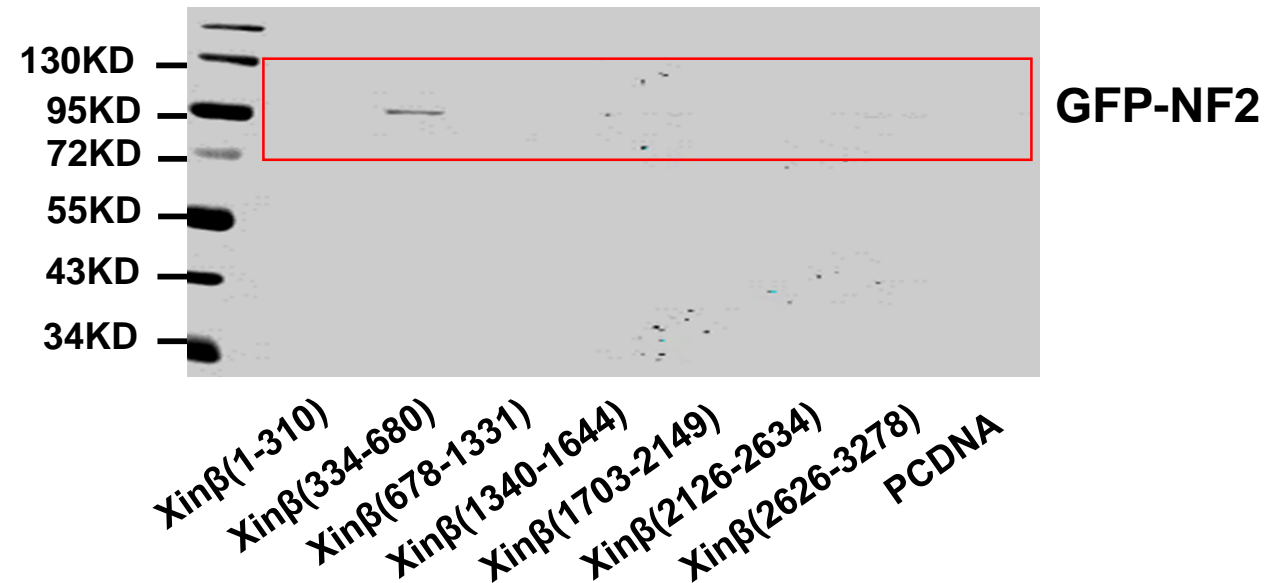

Fig 4g

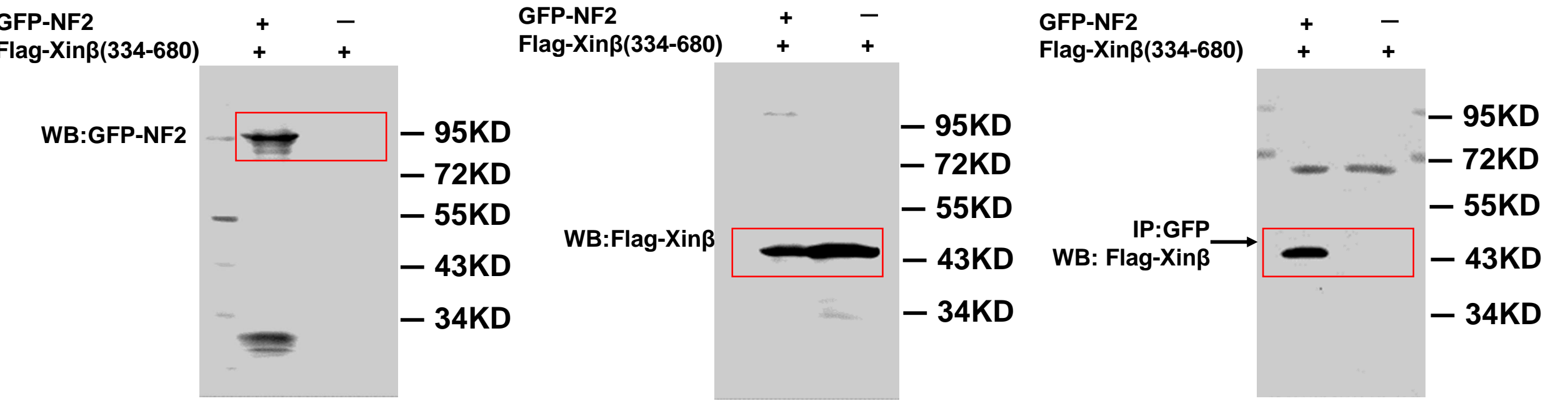

Fig 4h

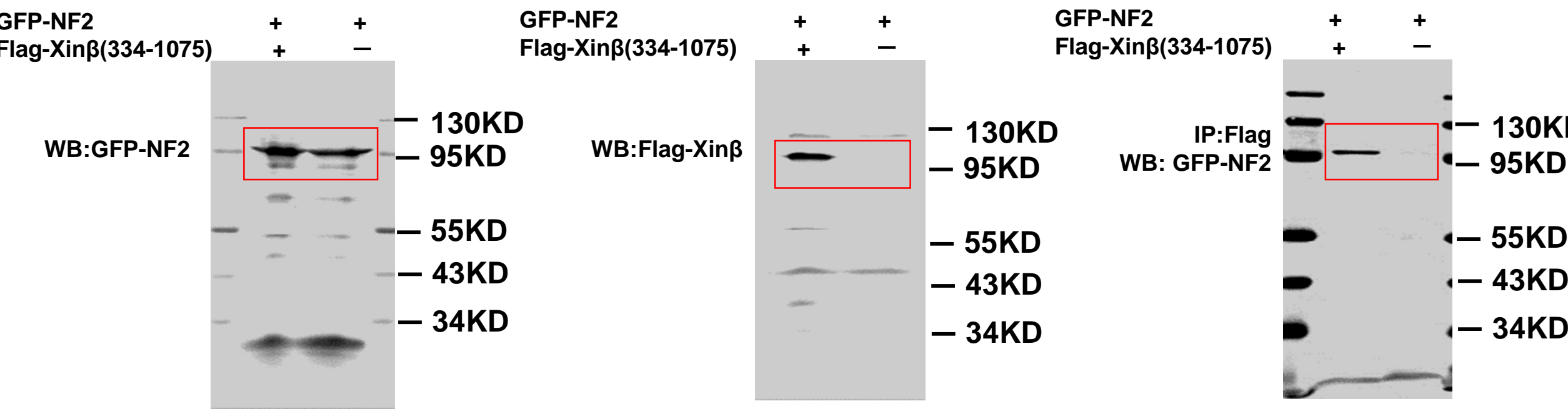

Fig 4h

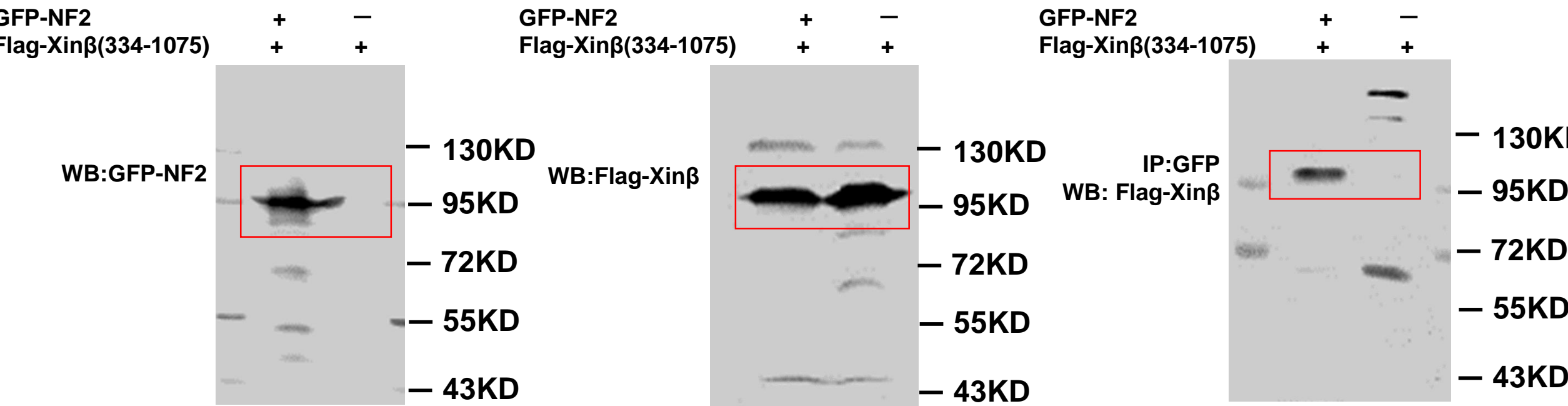

Fig 4j

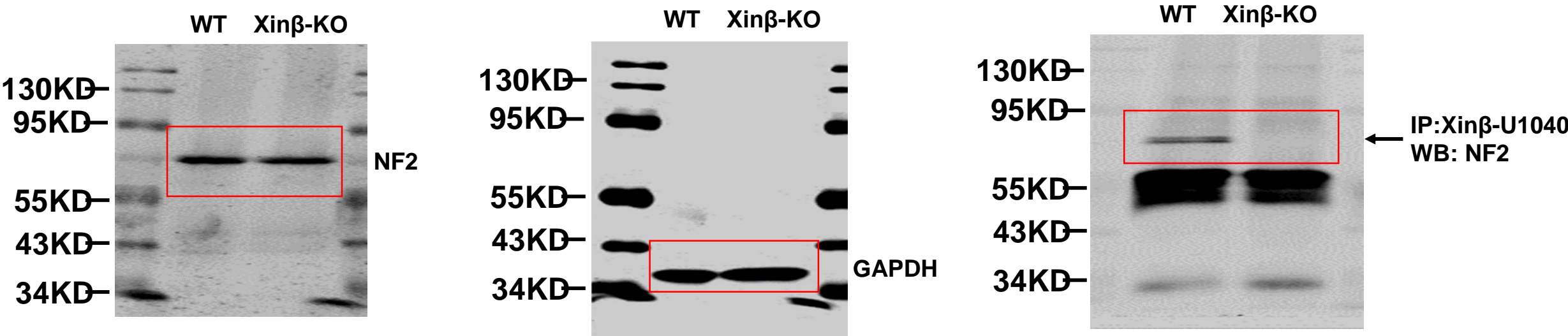

Supplement: Supplementary file 6 — Source Data [file 41467_2020_18379_MOESM6_ESM.zip › NCOMMS-20-07805B Western Blot Source Data.pdf]
